# Patient Stratification for Oral Transitional Therapy in Bacterial Endocarditis

**DOI:** 10.1093/cid/ciad194

**Published:** 2023-03-31

**Authors:** Clark D Russell

**Affiliations:** Centre for Inflammation Research, The Queen's Medical Research Institute, University of Edinburgh, Edinburgh, United Kingdom

To the Editor—Freling and colleagues [1] should be congratulated for the pragmatic implementation and thorough reporting of an oral therapy transition protocol for bacterial endocarditis. Application of clinical trial findings can be complicated by heterogeneity between different trials of the same disease, and differences in clinical characteristics of people recruited to trials compared with “real-life” patient cohorts [2]. Three randomized trials have investigated oral therapy for bacterial endocarditis, all indicating oral/partial oral therapy is safe and effective [3–5]. However, there are substantial differences in patient characteristics and oral antimicrobial regimes between these trials (Figure 1 and Supplementary Table 1).

**Figure 1.**
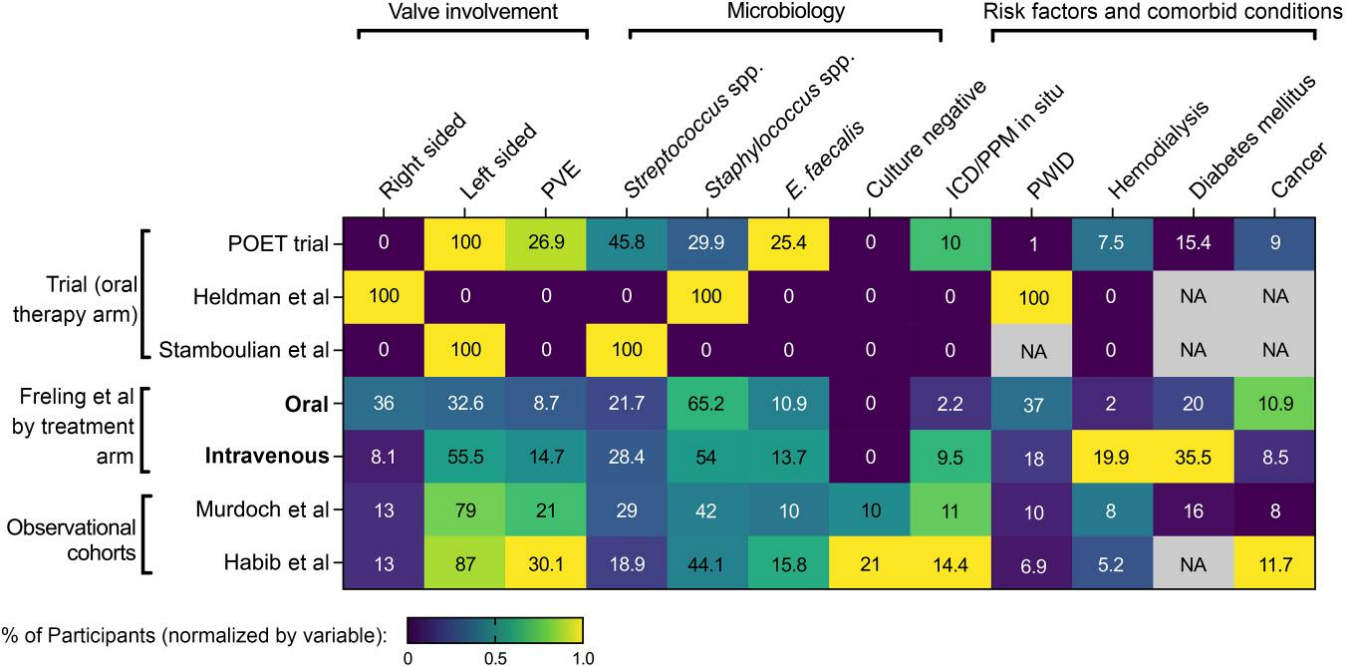
Cohort characteristics of clinical trials and observational studies of bacterial endocarditis. The heat map is shaded by normalized percentage for each variable (ie, column minimum to maximum), and the value inside each cell is the actual percentage from the study. For the 3 randomized trials (Partial Oral Treatment of Endocarditis [POET] trial [5], n = 201; Heldman et al [4], n = 45; Stamboulian et al [3], n = 15), data pertain to the oral therapy arm. Also shown are results for Freling et al [1], Habib et al [6], and Murdoch et al [7]; Habib et al reported *Enterococcus* spp., not *Enterococcus faecalis* specifically. Abbreviations: ICD, implantable cardiac defibrillator; PPM, permanent pacemaker; PVE, prosthetic valve endocarditis; PWID, person who injects drugs.

Suitability for oral transitional therapy in the protocol used by Freling et al [1] was not specifically restricted by patient characteristics, though differences in valve involvement, implantable cardiac defibrillator (ICD)/permanent pacemaker (PPM) presence, diabetes mellitus, and hemodialysis did emerge between the 2 groups (Figure 1). Left-sided valve involvement, prosthetic valve endocarditis, hemodialysis, and presence of an ICD/PPM were also less common in the oral transitional group than in observational cohorts, and patients were younger (Figure 1) [6, 7]. The findings are therefore not necessarily applicable to all people with bacterial endocarditis. Furthermore, although patient numbers (and the number of treatment failures) were too low to permit robust identification of factors associated with clinical success, numeric differences were apparent for people with end-stage renal failure, chronic obstructive pulmonary disease, liver disease, and cancer (lower success rates). No clear predictors of oral therapy failure were identified in the Partial Oral Treatment of Endocarditis (POET) trial either [5].

How can the subgroup of people with bacterial endocarditis most suitable for transitional oral therapy be defined? The protocol used to select patients in the study reported by Freling et al [1] is an invaluable starting point for applying this approach to clinical practice immediately. Of the 257 included patients, 46 (18%) had been identified as suitable for oral transitional therapy. The findings provide additional specific confidence when applying the approach to younger people with right-sided native valve endocarditis (especially people who inject drugs), people without end-stage renal failure requiring hemodialysis, liver disease or cancer, and people without an ICD/PPM in situ. Trial findings suggest that this could be an overly conservative approach, since people with differing characteristics (more similar to real-life cohorts) were recruited and successfully managed with oral/partial oral therapy (eg, approximately 25% with prosthetic valve endocarditis, approximately 10% with PPM/ICD in situ, and 7.5% receiving hemodialysis in the POET trial) (Figure 1). However, an important caveat to this is the lower mortality rate in clinical trial populations compared with real-life cohorts (Supplementary Figure 1; similar to findings in *Staphylococcus aureus* bacteremia [2]). Owing to selection biases introduced by eligibility criteria during recruitment, clinical trial populations can remain distinct from real-life despite similarities in key clinical characteristics.

Patient stratification for oral transitional therapy could include valve involvement, the presence or involvement of prosthetic material, and specific comorbid conditions. Additional variables requiring investigation include duration of intravenous lead-in (no impact on clinical success, even if ≤7 days [1]), local complications (eg, aortic root abscess), and coexistent infection foci (eg, discitis). Overall, factors associated with the success or failure of oral transitional therapy need to be defined to refine patient stratification for this approach. Ongoing investigation of the real-life implementation of clinical trial findings will be required to achieve this.

## Supplementary Data

Supplementary materials are available at *Clinical Infectious Diseases* online. Consisting of data provided by the authors to benefit the reader, the posted materials are not copyedited and are the sole responsibility of the authors, so questions or comments should be addressed to the corresponding author.

## Supplementary Material

ciad194_Supplementary_Data
