# Supplementary material for: Patient Stratification for Oral Transitional Therapy in Bacterial Endocarditis
Source: Clin Infect Dis. 2023 Mar 31;77(3):494–5. doi: 10.1093/cid/ciad194 (PMC10425185; doi:10.1093/cid/ciad194)
Supplement: ciad194_Supplementary_Data [file ciad194_supplementary_data.pdf]

Patient stratification for oral transitional therapy in bacterial endocarditis

Supplementary Table 1: Oral regimes studied in bacterial endocarditis

Study	Year published	Total n	IV n	PO n	Oral/partial oral regime
**Randomised trial**					
**Stamboulian *et al*[1]**	1991	30	15	15	Amoxicillin (2w) following 2w ceftriaxone.
**Heldman *et al*[2]**	1996	44	25	19	Ciprofloxacin plus rifampicin (4w)
**Iversen *et al* (POET)[3]**	2019	400	199	201	Various combination-based regimes (mainly beta-lactam plus rifampicin or moxifloxacin). Median IV lead-in 17d.
**Real-world application**					
**Freling *et al*[4]**	2023	257	211	46	Various beta-lactam, fluroquinolone, linezolid or co-trimoxazole based regimes as monotherapy or combination (usually plus rifampicin).<br>Linezolid monotherapy used in 26/46 cases.<br>Median IV lead-in 16d; median PO transitional therapy 28d.

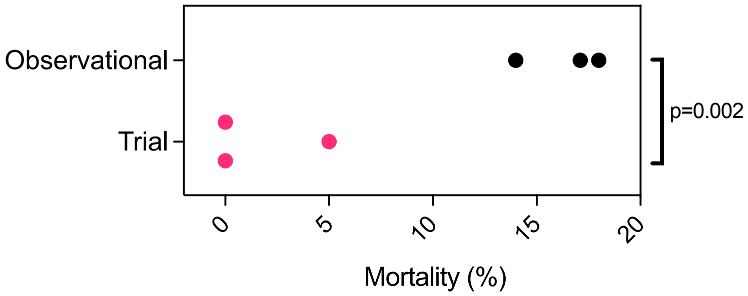

Supplementary Figure 1: Comparison of mortality between observational studies and clinical trials of oral therapy in bacterial endocarditis.
Percentages compared using unpaired t-test.

Observational

Habib *et al*[5]	17.1% (in-hospital)
Murdoch *et al*[6]	18% (in-hospital)
Freling *et al**[4]	14% (last follow-up)

*Trial**

Iversen *et al* (POET)[3]	5% (6 months)
Heldman *et al*[2]	0%
Stamboulian *et al*[1]	0%

*combined PO and IV arms

References

1. Stamboulian D, Bonvehi P, Arevalo C, et al. Antibiotic management of outpatients with endocarditis due to penicillin-susceptible streptococci. *Rev Infect Dis.* 1991;13 Suppl 2:S160-163.
2. Heldman AW, Hartert TV, Ray SC, et al. Oral antibiotic treatment of right-sided staphylococcal endocarditis in injection drug users: prospective randomized comparison with parenteral therapy. *Am J Med.* 1996;101(1):68-76.
3. Iversen K, Ihlemann N, Gill SU, et al. Partial Oral versus Intravenous Antibiotic Treatment of Endocarditis. *New England Journal of Medicine.* 2018;380(5):415-424.
4. Freling S, Wald-Dickler N, Banerjee J, et al. Real-world Application of Oral Therapy for Infective Endocarditis: A Multicenter Retrospective, Cohort Study. *Clin Infect Dis.* 2023.
5. Habib G, Erba PA, Iung B, et al. Clinical presentation, aetiology and outcome of infective endocarditis. Results of the ESC-EORP EURO-ENDO (European infective endocarditis) registry: a prospective cohort study. *Eur Heart J.* 2019;40(39):3222-3232.
6. Murdoch DR, Corey GR, Hoen B, et al. Clinical presentation, etiology, and outcome of infective endocarditis in the 21st century: the International Collaboration on Endocarditis-Prospective Cohort Study. *Arch Intern Med.* 2009;169(5):463-473.
